# The stability of the coiled-coil structure near to N-terminus influence the heat resistance of harpin proteins from *Xanthomonas*

**DOI:** 10.1186/s12866-020-02029-6

**Published:** 2020-11-12

**Authors:** Yue Liu, Xiaoyun Zhou, Wenbo Liu, Weiguo Miao

**Affiliations:** 1 College of Plant Protection, Hainan University, Haikou, Hainan Province China; 2 Key Laboratory of Green Prevention and Control of Tropical Plant Diseases and Pests (Hainan University), Ministry of Education, Haikou, Hainan Province China

**Keywords:** *Xanthomonas*, Harpin protein, Coiled-coil structure, Heat resistance, Hypersensitive response

## Abstract

**Background:**

Heat resistance is a common characteristic of harpins, a class of proteins found in Gram-negative bacteria, which may be related to the stability of coiled-coil (CC) structure. The CC structure is a ubiquitous protein folding and assembly motif made of α-helices wrapping around each other forming a supercoil. Specifically, whether the stability of the CC structure near to N-terminus of four selected harpin proteins from *Xanthomonas* (hereafter referred to as Hpa1) would influence their characteristics of heat resistance was investigated. We used bioinformatics approach to predict the structure of Hpa1, used the performance of hypersensitive response (HR)-induction activity of Hpa1 and circular dichroism (CD) spectral analyses to detect the relationship between the stability of the CC structure of Hpa1 and heat resistance.

**Results:**

Each of four-selected Hpa1 has two α-helical regions with one in their N-terminus that could form CC structure, and the other in their C-terminus that could not. And the important amino acid residues involved in the CC motifs are located on helices present on the surface of these proteins, indicating they may engage in the formation of oligo mericaggregates, which may be responsible for HR elicitation by harpins and their high thermal stability. Increased or decreased the probability of forming a CC could either induce a stronger HR response or eliminate the ability to induce HR in tobacco after high temperature treatment. In addition, although the four Hpa1 mutants had little effect on the induction of HR by Hpa1, its thermal stability was significantly decreased. The α-helical content increased with increasing temperature, and the secondary structures of Hpa1 became almost entirely α-helices when the temperature reached 200 °C. Moreover, the stability of the CC structure near to N-terminus was found to be positively correlated with the heat resistance of Hpa1.

**Conclusions:**

The stability of the CC structure might sever as an inner drive for mediating the heat resistance of harpin proteins. Our results offer a new insight into the interpretation of the mechanism involved in the heat resistance of harpin protein and provide a theoretical basis for further harpin function investigations and structure modifications.

**Supplementary Information:**

The online version contains supplementary material available at 10.1186/s12866-020-02029-6.

## Background

Proteins could lose their functional activities under various stress conditions, mainly due to the destruction of their active three-dimensional (3-D) structure [1]. However, some proteins, including harpins, can maintain their functional activity under heat-stressed conditions [2–4]. Harpins are a class of proteins encoded by *hrp* genes in Gram-negative bacteria and are secreted by the type III secretion system during interactions between pathogens and plants [5]. The first harpin was originally isolated from *Erwinia amylovora* [6] and subsequently, nearly two dozen harpins have been found and characterized in the following plant-infecting bacteria: *Xanthomonas*, *Pseudomonas*, *Erwinia*, and *Ralstonia* [2, 4, 6–12]. To date, most research on harpins has mainly focused on the defining of their roles in plants [3, 13–17]. It was found that harpin proteins not only can act as virulence factors (or translocators) for bacterial pathogens, but also can act as elicitors to induce hypersensitive response (HR) [3, 15, 18–20], systemic-acquired resistance [21], plant growth and yield [3, 22], insect resistance [16], and drought tolerance [23]. Harpins have a common characteristic, heat resistance, which have been reported to maintain their HR-elicitor activity after 10 min (min) treatment at a high temperature of 100 °C in tobacco plants [2–4]. However, few studies had attempted to elucidate the mechanism responsible for their heat resistance. At present, the reasons that affect the heat-resistant properties of Harpin protein were summarized as follows. One is that the lack of obvious 3-D structure formed by the disulfide bonds of Cys residues may affect the thermal stability of harpins, however, harpins lack Cys residues [5]. Another one is that a coiled-coil (CC) structure forming at N-terminal α-helical region in HpaXcm from *X. citri* pv. *mangiferaeindicae* [2] might attribute to the thermal stability of Hapin. However, the factors affect the heat resistance of harpin protein is still unclear.

The canonical CC domain is a typical motif commonly found in many structural and/or regulatory proteins with important functions ranging from macromolecular complex formation to molecular recognition [24]. The CC motif consists of two or more right-handed amphipathic α-helices winding around one another to form helical bundles with left-handed supercoils [7, 24, 25]. CC motif sequences are characterized by a heptad repeat of seven aa residues at positions *a*, *b*, *c*, *d*, *e*, *f*, and *g*. The aa residues at the *a* and *d* positions in the CC motif are preferentially nonpolar hydrophobic residues (i.e., leucine, isoleucine, and valine) and are specifically located within CC structures [7, 24]. The aa residues at the *e* and *g* positions are preferentially polar residues (i.e., lysine and glutamate) and located outside the hydrophobic core formed through interactions between the two aa residues at the *a* and *d* positions [7, 24]. Ji et al. (2011) has demonstrated that mutating amino acids at *a, d, e* or *g* positions could increase or decrease the probability of forming a CC (PFC) by [24]. In general, the aa residues at the *a*, *d*, *e*, and *g* positions which can influence the PFC have an important effect on the stability of the CC structure [26–28]. In addition, temperature, solution composition, pH, length of helical chain, and the number of helices in the proteins have some influences on the stability and structural specificity of CC structure [25, 29–31].

The missense mutants of harpin proteins could destroy the α-helical or CC region, which result in the elimination or reduction the HR elicitation activity [3, 10, 24, 32]. For example, Kim et al. (2004) showed that the single missense and double missense harpin mutants of *X. axonopodis* pv. *glycines* that are predicted to be an α-helix abolished, which leading to the reduction of the HR elicitation activity in tobacco [10]. Wang et al. (2008) showed that deletion of codons for 12 highly hydrophilic amino acids that partially overlap the N-terminal α-helical regions of harpin proteins of *X. oryzae* pathovars was critical for the elicitation of HR in tobacco. Furthermore, the single missense mutant of *X. oryzae* pathovars that are predicted to destroy the coiled-coil integrity and inhibit the dimer formation could eliminate the HR elicitation activity in tobacco [32]. Ji et al. (2011) found that wild type peptide N14 with higher possibility of forming a CC had the ability to induce HR in tobacco, while its mutational peptide N14-L1S with little possibility of CC information eliminated HR activity in tobacco by generating the mutant of the N- and C-terminal peptides of Hpa1Xoo of *X. oryzae* pv. *oryzae* [24]. Previously, we found that the fragment of HpaXm of *X. citri* subsp. *malvacearum* is predicted to be important for the formation of α-helix and is sufficient to induce the HR in tobacco [3]. Overall, it is well known that the N-terminal CC region of harpin protein is essential for eliciting HR. All previous studies mainly focused on the influence of N-terminal CC region of harpin protein in the HR elicitation activity, however, the relationship between the CC structure and the thermal stability of harpin remians unclear.

In this study, we intend to explore the factors affecting the heat resistance of harpin proteins by exploring the relationship between the stability of CC structure and the heat resistance of four selected harpin proteins. The four harpins (hereafter referred to as Hpa1) from *Xanthomonas* were selected as subjects, which were HpaXm, Hpa1Xoo, HpaXpm, and HpaXcm, respectively identified from the cotton leaf blight bacteria, *Xanthomonas citri* subsp. *malvacearum* (*Xm*) [4]; the rice bacterial leaf blight bacteria, *X. oryzae* pv. *oryzae* (*Xoo*) [33]; the cassava blight bacteria, *X. phaseoli* pv. *manihotis* (*Xpm*) [34]; and, the bacterial black spot of mango bacteria, *X. citri* pv. *mangiferaeindicae* (*Xcm*) [2]. For the selected proteins, the secondary and spatial structure were predicted, and the ability on HR induction and the helical content in secondary structure were monitored treated with temperatures ranging from 28 °C to 200 °C.

## Methods

### Sources of bacterial

Strains of *Xm*, *Xpm*, and *Xcm* were identified by the Key Laboratory of Green Prevention and Control of Tropical Plant Diseases and Pests (Hainan University), Ministry of Education, Haikou, Hainan Province, China. The strain of *X. oryzae* pv. *oryzae* (*Xoo*) used in this study was provided by Dr. Gong-you Chen (School of Agriculture and Biology, Shanghai Jiao Tong University, and Key Laboratory of Urban (South) Ministry of Agriculture of China, Shanghai, China). All bacterial strains and the expression vector pGEX6p-1 were maintained in the laboratory until use. Before use, strains of *Xm*, *Xoo*, *Xpm*, and *Xcm* were cultured individually in nutrient broth liquid or nutrient broth agar plates at 28 °C as previously described [2].

### Construction of wild-type and mutant strains

Full length sequences of *HpaXm*, *Hpa1Xoo*, *HpaXpm*, and *HpaXcm* were PCR-amplified from the bacterial strains of *Xm*, *Xoo*, *Xpm*, and *Xcm*, respectively. PCR primers with specific restriction sites (Table 1) were designed based on the published sequences of *HpaXm* (ABG36696.1), *Hpa1Xoo* (ACD56757.1), *HpaXpm* (ATB17313.1), and *HpaXcm* (ATB17312.1). PCR reactions were performed using a TransStart FastPfu Fly DNA Polymerase Kit (TransGen Biotech, Beijing, China) as follows: pre-denaturation at 95 °C for 2 min, followed by 35 cycles at 95 °C for 20 s, 58 °C for 20s, and 72 °C for 1 min, and a final 10 min extension at 72 °C. The resulting *HpaXm*, *Hpa1Xoo*, *HpaXpm*, and *HpaXcm* products were double digested with restriction enzymes, either *BamH*I and *Not*I, or *BamH*I and *Xho*I, and then were inserted individually into the *BamH*I/*Not*I or *BamH*I/*Xho*I site in vector pGEX6p-1, carrying a T7 promoter and a GST tag. The recombinant pGEX6p-1 vectors were sequenced and then transformed individually into *Escherichia coli* strain BL21 (DE3).

**Table 1:** Gene names, gene accession numbers and primers used in this study

Name	Accession number		Primer sequence (5′-3′)	PCR product size (bp)	Purpose
hpaXm	DQ643828.1	Forward	CGGGATCCATGAATTCTTTGAACACACAG (BamHI)^a^	428	PCR for cloning
		Reverse	AAGGAAAAAAGCGGCCGCTTACTGCATCGATCCGGTGTCGCT (NotI)^a^		
hpa1Xoo	CP000967.2	Forward	CGGGATCCATGAATTCTTTGAACACACAA (BamHI)^a^	446	PCR for cloning
		Reverse	AAGGAAAAAAGCGGCCGCTTACTGCATCGATGCGCTGTCGCT (NotI)^a^		
hpaXpm	KY765410.1	Forward	CGGGATCCATGAACCCAGCGGCGCAGACC (BamHI)^a^	435	PCR for cloning
		Reverse	CCGCTCGAGTTACTGCATCGATCCGGTGTCGCT (XhoI)^a^		
hpaXcm	KY697778.1	Forward	CGGGATCCATGATGAATTCTTTGAACACA (BamHI)^a^	422	PCR for cloning
		Reverse	CCGCTCGAGTTACTGCATCGATCCGGTGTCGCT (XhoI)^a^		

^a^The underlined sequences are restriction site BamHI, NotI or XhoI

Based on the predicted CC structures and the key aa residual sites, primers for selected mutation sites were designed (Table 2). Site-directed mutagenesis of HpaXm, Hpa1Xoo, HpaXpm, and HpaXcm were performed using a Fast Site-Directed Mutagenesis (FDM) kit according to the manufacturer’s instructions (TIANGEN, Beijing, China). Plasmids of pGEX6p-GST-Hpa1 were used for PCR reactions using specific primers (Table 2). PCR reactions were performed as follows: pre-denaturation at 95 °C for 2 min, followed by 18 cycles at 94 °C for 20 s, 55 °C for 10 s, and 68 °C for 2.5 min followed by a 5 min final extension at 68 °C. The resulting PCR products (50 μL each) were digested with 1 μL of *Dpn*I restriction enzyme for 1 h at 37 °C. The FDM competent cells were stored at − 80 °C until use. The cells were thawed on ice immediately prior to use and then were transformed with 5 μL *Dpn*I-digested PCR reaction product. All the genes cloned into plasmids were fully sequenced prior to being transformed into BL21 cells.

**Table 2:** Primers used for hpa1 deletion mutant constructions

Primer names	Primer sequence (5′-3′)	Purpose
HpaXmΔL39A-F	CATCTCGGAAAAGCAGGCCGACCAGCTGCTGACCC	Primers used to construct hpaXmΔL39A
HpaXmΔL39A-R	GGGTCAGCAGCTGGTCGGCCTGCTTTTCCGAGATG	
Hpa1XooΔD41A-F	CTCGGAAAAGCAACTGGCTCAGTTGCTGTGCCAGC	Primers for constructing hpa1XooΔD41A
Hpa1XooΔD41A-R	GCTGGCACAGCAACTGAGCCAGTTGCTTTTCCGAG	
HpaXpmΔM54A-F	ACACAGCTCATCATCCTGGCCCTGCTGC	Primers for constructing hpaXamΔM54A
HpaXpmΔM54A-R	GGATGATGAGCTGTGTCAGCAACTGGTC	
HpaXcmΔL40A-F	CATCTCGGAAAAGCAAGCCGACCAGCTGCTGACCC	Primers for constructing hpaXcmΔL40A
HpaXcmΔL40A-R	GGGTCAGCAGCTGGTCGGCTTGCTTTTCCGAGATG	

### Protein preparation

The transformed *E. coli* was cultured at 37 °C in an LB medium plate with 100 μg/mL ampicillin as previously described [4]. When the OD600 of bacterial cell culture reached 0.8, the temperature was decreased to 28 °C to induce the expressions of individual cloned genes by adding 0.5 mM isopropyl-β-D-thiogalactoside. Four hours (h) later, bacterial cells in each culture were harvested and pelleted by centrifugation at 3500 *g* for 5 min. The cell pellet was resuspended in phosphate buffered saline (pH 7.2), and then was ruptured with ultrasound. After 5 min centrifugation at 3500 *g*, the supernatant containing soluble proteins and the sediment containing insoluble proteins were respectively loaded into wells in 12% SDS-PAGE gels as previously described [2]. Subsequently, to determine the expression efficiency of each fusion protein, the separated proteins were transferred onto polyvinylidene fluoride membranes with a polyclonal antibody against GST and a goat anti-rabbit lgG-HRP conjugated antibody. Totally, four wild-type proteins, HpaXm, Hpa1Xoo, HpaXpm, and HpaXcm, and the four mutant proteins, HpaXmΔL39A (Leu at position 39 is replaced by Ala), Hpa1XooΔD41A (Asp at position 41 is replaced by Ala), HpaXpmΔM54L (Met at position 54 is replaced by Leu), and HpaXcmΔL40A (Leu at position 40 is replaced by Ala) with GST tags (GST-Hpa1) were prepared. The proteins were heat-treated in a water bath or an oil bath at 28 °C, 40 °C, 60 °C, 80 °C, 100 °C, 150 °C, or 200 °C. After 10 min of heat treatment, the protein solutions were centrifuged at 3500 *g* for 10 min, and then the supernatant soluble, GST-Hpa1, were collected and adjusted to a consistent concentration for subsequent experiments. GST-Hpa1 was used for HR assays and circular dichroism (CD) measurements with GST protein as a control.

### Plant growth and treatments

Tobacco (*Nicotiana tabacum* cv. Samsun-NN) seeds were stored in the laboratory at 4 °C [3]. The seeds were grown in soil in 10 cm pots in a growth chamber set at 25 °C. The HR assay was performed by injecting 10 μM GST-Hpa1 into leaves of 30-day-old tobacco seedlings as previously described [3]. The HR elicited in leaves was scored at two days post injection of 10 μM GST-Hpa1. The activity of individual Hpa1 was determined based on the ratio between the size of the necrotic lesion caused by the HR and the injection site, as previously described [2]. The size of the HR or the injection site was estimated using ImageJ software. Fifteen plants were used for each treatment, and each experiment was repeated three times.

### Circular dichroism (CD) spectra

CD spectra were measured using a Jasco-810 spectropolarimeter (Jasco Co., Japan) equipped with a Peltier thermoelectric temperature control system and a flow-through HPLC cell, as previously described [13, 24, 31]. The whole process was controlled using Jasco Spectra Manager software (Jasco Co., Japan). Scans were performed in a 0.1 cm pathlength quartz cell with a bandwidth of 1.0 nm at 20 °C from wavelength 190 to 280 nm at 0.1 nm intervals and 100 nm/min. The scan response time was set at 0.25 s per scan. The concentrations of GST-Hpa1 and GST proteins for each sample were adjusted to 1 mg/mL. The far-ultraviolet CD spectrum was determined based on the mean of three measurements after subtraction of the buffer background. The curves represented the results from triplicate measurements. After baseline correction, the ellipticities in mdeg were converted to molar ellipticities (deg cm^2^/dmol) by normalizing peptide bond concentrations. The proportions of different types of secondary structure were estimated using Jasco Spectra Manager software.

### Bioinformatics

Protein CC structures were predicted using PRABI software (https://npsa-prabi.ibcp.fr). Secondary structures prediction for four Hpa1 using NPS (https://npsa-prabi.ibcp.fr/cgi-bin/secpred_hnn.pl). The 3-D structures were predicted based on the corresponding amino acid sequences of the four proteins (HpaXm ABG36696.1, Hpa1Xoo ACD56757.1, HpaXpm ATB17313.1, and HpaXcm ATB17312.1) by the I-TASSER server (https://zhanglab.ccmb.med.umich.edu/I-TASSER/, and obtained 3D structures were modified using PyLOM software.

### Statistical analyses

Statistical analyses were performed using the Statistical Program for Social Science 17.0 software. Data were presented as means ± the standard deviation from at least three experiments. The ANOVA (Bonferroni) method was used to determine statistical differences between treatments (*, *p* < 0.05; **, *p* < 0.01).

## Results

### Prediction of the structure of Hpa1

**Fig. 1:**
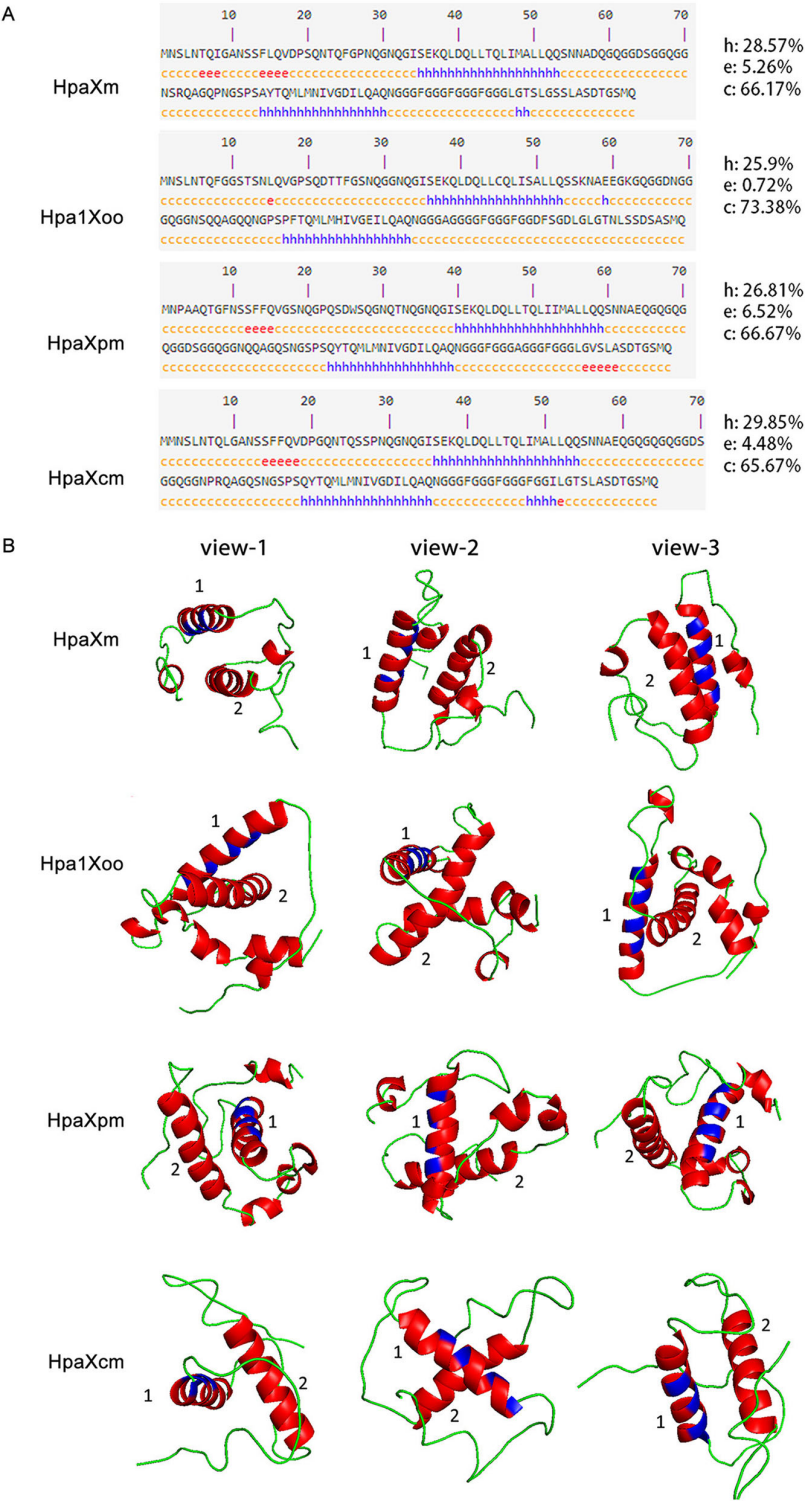
Secondary structure prediction and three-dimensional (3-D) structure prediction of the four Hpa1. **a** Secondary structures prediction for four Hpa1 using NPS (https://npsa-prabi.ibcp.fr/cgi-bin/secpred_hnn.pl). Secondary structure predictions indicated the presence of α-helix (h), extended strand (e), and random coil (c) structures in the four proteins. **b** The 3-D structures were predicted by the I-TASSER server (https://zhanglab.ccmb.med.umich.edu/I-TASSER/). Models of the 3D structures were modified using PyLOM software. Ribbon representations of the possible 3-D structures of four Hpa1 from three different views. Helical motifs are highlighted in red, while loop and turn motifs are highlighted in green. Sheet motifs are highlighted in yellow. Residues involved in the CC motifs are highlighted in blue. The two numbers ‘1’ and ‘2’ represent two stretches. Stretch 1 is a predicted α-helical region at the N-terminus; stretch 2 is a predicted α-helical region at the C-terminus

In order to explore the relationship between harpin protein structure and heat resistance, the secondary structure of the four Hpa1 were predicted and compared. The prediction results showed that each of the four Hpa1 has one α-helical region in their N-terminus and one in their C-terminus (Fig. 1a). HpaXm comprised 28.57% α-helices, 5.26% extended strand, and 66.17% random coil; Hpa1Xoo comprised 25.9% α- helices, 0.72% extended strand, and 73.38% random coil; HpaXpm comprised 26.81% α- helices, 6.52% extended strand, and 66.67% random coil; and HpaXcm comprised 29.85% α- helices, 4.48% extended strand, and 65.67% random coil. Overall, except for random coil, the α-helical content in each protein sequence accounts for the highest proportion.

To further verify the formation of the CC structure of the four Hpa1, their possible 3-D structures were predicted. Consistent with the results obtained from the secondary structure, each protein is composed predominantly of two major helical regions (i.e., HpaXm stretch-1 residues 36–52, stretch-2 residues 84–101; Hpa1Xoo stretch-1 residues 37–53, stretch-2 residues 87–101; HpaXpm stretch-1 residues 41–57, HpaXpm stretch-2 residues 93–109; HpaXcm stretch-1 residues 37–53, stretch-2 residues 89–106) connected by turns and loops (Fig. 1b). The probability of forming a CC predictions (PFC) indicated that Leu39, Leu42, Leu46, and Ala49 in HpaXm, Leu40, Leu43, Leu47, and Ala50 in Hpa1Xoo, Leu44, Leu47, Met54, and Leu51 in HpaXpm, and Leu40, Leu43, Leu47 and Ala50 in HpaXcm are crucial amino acids for the formation of the CC structure. The predicted 3-D structures showed that the amino acid residues involved in the CC motifs are located on helices present on the surface of these proteins, and these motifs which can take part in the formation of oligo mericaggregates and may be responsible for their heat resistance. (Fig. 1b).

### The production of Hpa1 mutant proteins without loss of HR-induction activity that predicted to increase or decrease the PFC

**Fig. 2:**
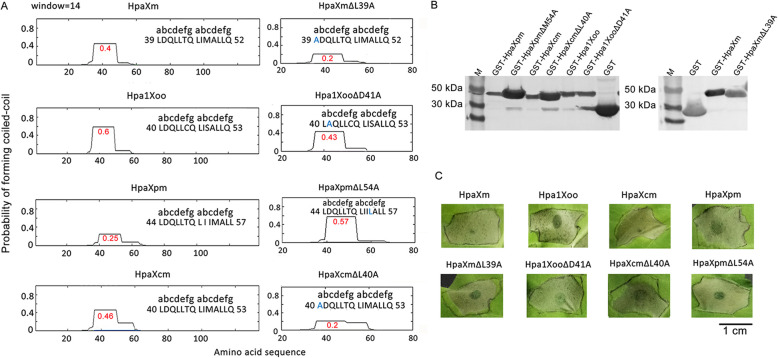
Predictions of the PFC of Hpa1 and mutants, and detection their ability to induce HR. **a** Predictions of PFC in Hpa1 and mutants. Letters a - g designate the position of residues in the heptad repeats. The PFC was obtained by scanning windows of 14 residues using the MTIDK matrix. Amino acid residues that were replaced are shown in blue. The PFC value for each of the proteins is shown in red. **b** Western blot analysis of efficient expression of Hpa1, and their mutants. Wild-type Hpa1, their mutants and GST (negative control) were probed with a polyclonal antibody specific for GST and then a goat anti-rabbit lgG-HRP antibody. The induced bacterial cultures were ultra-sonicated followed by centrifugation. The resulting supernatants were used as soluble protein samples. M, molecular markers. Molecular mass marker in size (kDa) is indicated on the left-hand side. **c** The abilities of Hpa1 and mutants to induce HR. Tobacco leaves infiltrated with 10 μM Hpa1 or their mutant proteins. The size of the injection site is indicated by a solid black line

To investigate the relationship between the heat resistance and the PFC of harpin proteins, the PFC of Hpa1 and mutant proteins were predicted. The higher the PFC, the favor the formation of the CC structure formats, and the stronger the stability of the CC structure shapes. The predicted CC regions in the four Hpa1 were HpaXm 39-LDQLLTQ-LIMALLQ-52 with a 0.4 PFC, Hpa1Xoo 40-LDQLLCQ-LISALLQ-53 with a 0.6 PFC, HpaXpm 44-LDQLLTQ-LIIMALL-57 with a 0.25 PFC, and HpaXcm 40-LDQLLTQ-LIMALLQ-53 with a 0.46 PFC (Fig. 2a). The hydrophobic leucine residue of the mutant HpaXmΔL39A (39-ADQLLTQ-LIMALLQ-52) was replaced by a hydrophobic alanine residue at the *a* position in the first heptad. Analysis of this mutant showed that the PFC of HpaXmΔL39A was decreased to 0.2 (Fig. 2a). The PFC of the mutant Hpa1XooΔD41A (40-LAQLLCQ-LISALLQ-53), which had a hydrophilic aspartic acid residue replaced by a hydrophobic alanine residue at the *b* position in the first heptad, was decreased to 0.43. The PFC of mutant HpaXpmΔM54L (44-LDQLLTQ-LIILALL-57), which had a hydrophilic methionine residue replaced by a hydrophobic leucine residue at the *d* position in the second heptad, was increased to 0.57. The PFC of the mutant HpaXcmΔL40A (40-ADQLLTQ-LIMALLQ-53), which had a hydrophobic leucine residue replaced by a hydrophobic alanine residue at the *a* position in the first heptad, was decreased to 0.2 (Fig. 2a). The effective expression of four Hpa1 and their mutanted proteins HpaXmΔL39A, Hpa1XooΔD41A, HpaXpmΔM54L, and HpaXcmΔL40A were identified by Western blot. As expected, the results showed that the sizes of the mutant proteins are the same as their corresponding wild-type proteins as the amino acid sequence of each mutant protein only replaces one amino acid, and they all form a single band of molecular mass ~ 40 kDa, respectively (Fig. 2b and Additional file 1). These results indicated that all four Hpa1 and their mutanted proteins could be expressed efficiently. To further verify their ability to induce HR, tobacco leaves from different strains were respectively infiltrated with an aqueous solution of them and each of them displayed a strong HR (Fig. 2c. Overall, the four mutated proteins, HpaXmΔL39A, Hpa1XooΔD41A, HpaXpmΔM54L, and HpaXcmΔL40A, predicted to enable decrease/increase PFC that still have the ability to induce HR, indicating that they were suitable as the research object.

### The PFC of Hpa1 was positively correlated with their heat resistance

**Fig. 3:**
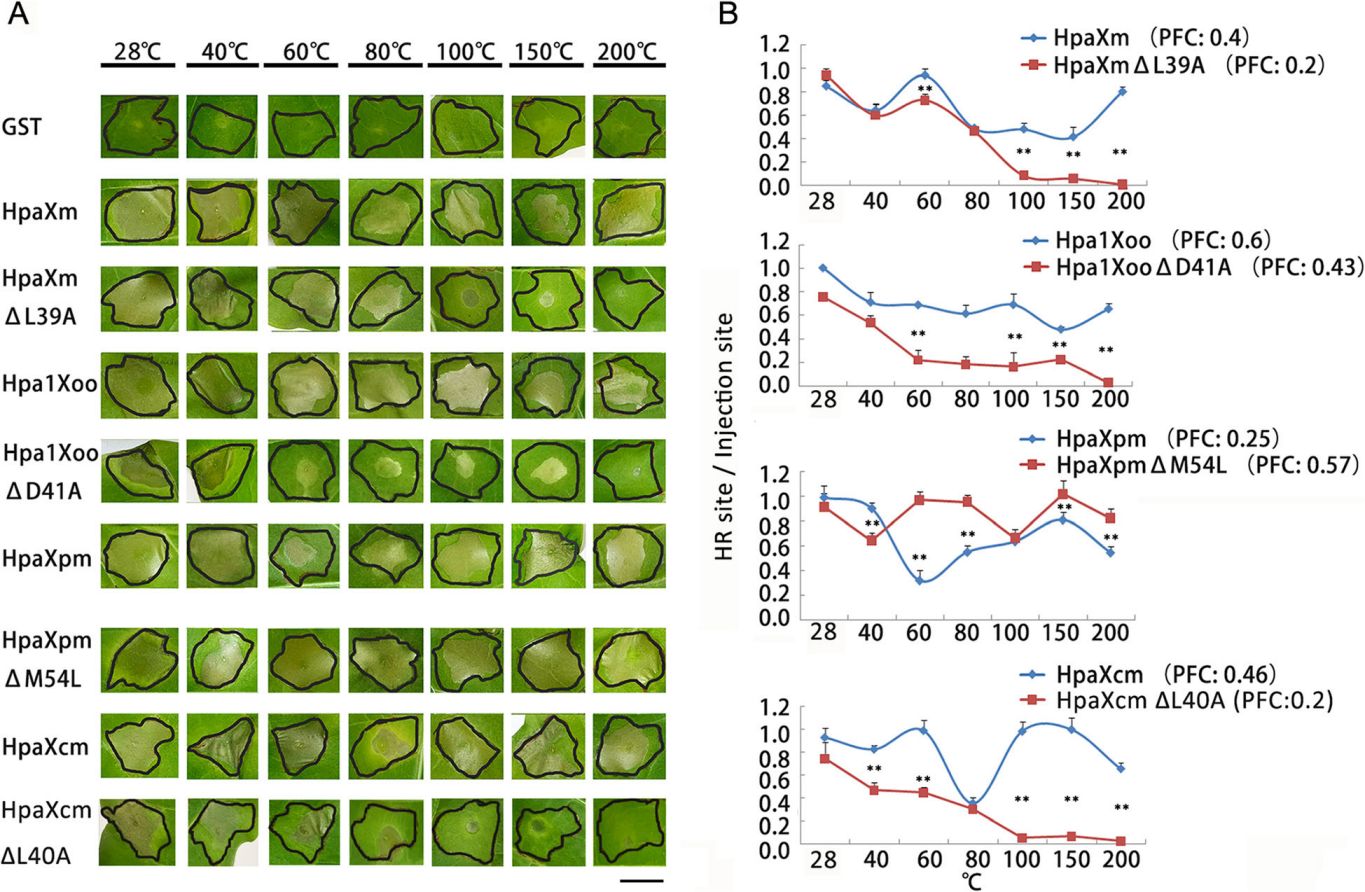
Effects of heat treatments on the abilities of Hpa1 and their mutants to induce HR. **a** Tobacco leaves infiltrated with 10 μM heat- or non-heat-treated Hpa1 or their mutant proteins. The size of the injection site is indicated by a solid black line. **b** The ability of each protein to induce HR was determined by the ratio between the size of the necrotic lesion caused by the HR and the size of the injection site. The values in y-axis indicate the means of three biological replicates per treatment. Error bars indicate the standard deviation of the mean (*n* = 3). Asterisks indicate a significant difference between the ability of the four Hpa1 to induce HR after treatment at different temperatures and that of their mutants, which obtained by one-way ANOVA (Bonferroni method, *P < 0.05, **P < 0.01)

To explore the effect of PFC on the thermal stability of Hpa1, the ability of the four Hpa1 to induce HR after treatment at different temperatures was compared to that of their mutants (Fig. 3). HR ratios (i.e., the ratio of the necrotic lesion caused by the HR to the injection site) induced by 60 °C-, 100 °C-, 150 °C-, or 200 °C-treated HpaXmΔL39A/Hpa1XooΔD41A/ HpaXcmΔL41A were significantly (*P* < 0.05) lower than those induced by HpaXm/Hpa1Xoo/ Hpa1Xoo treated at these temperatures. However, HR ratios induced by 60 °C-, 80 °C-, 150 °C-, or 200 °C-treated HpaXpmΔM54L were significantly higher (*P* < 0.05) than those induced by HpaXpm treated at these temperatures. The four Hpa1 proteins HpaXm, Hpa1Xoo, HpaXpm, and HpaXcm could still stimulate strong HR even at 200 °C. However, the mutanted proteins HpaXmΔL39A and HpaXcmΔL41A with reduced PFC could solely cause weakened HR at 100 °C and at 60 °C, respectively, while the mutanted protein HpaXpmΔM54L with increased PFC could still stimulate strong HR at 200 °C, which is even higher than wild-type HpaXpm. These results showed that the PFC of harpin protein increases with increasing heat-resistant ability, while the PFC decreases, with their decreasing heat-resistant ability. Therefore, the PFC of hpa1 is positively correlated with their heat resistance.

Specifically, the HR ratio induced by 200 °C-treated HpaXm was similar to that induced by 28 °C-treated HpaXm. However, HR ratios induced by 100 °C-, 150 °C-, or 200 °C-treated HpaXmΔL39A were lower than that of 28 °C-treated HpaXmΔL39A. The 100 °C- and 150 °C-treated HpaXmΔL39A only induced a slight HR around the injection hole in tobacco. The 200 °C-treated HpaXmΔL39A did not induce HR in tobacco. The HR ratio induced by 200 °C-treated Hpa1Xoo was even greater than that induced by 40 °C-treated Hpa1XooΔD41A. The 60 °C-, 80 °C-, 100 °C-, and 150 °C-treated Hpa1XooΔD41A only induced a slight HR around the injection hole in tobacco. The 200 °C-treated Hpa1XooΔD41A did not induce HR in tobacco. The HR ratio induced by 200 °C-treated Hpa1Xcm was even greater than that induced by 40 °C-treated HpaXcmΔL41A. The 100 °C- and 150 °C-treated HpaXcmΔL41A only induced a slight HR around the injection hole in tobacco. The 200 °C-treated HpaXcmΔL41A did not induce HR in tobacco.

### The increase in temperature makes the higher α-helical content in Hpa1 secondary structures

**Fig. 4:**
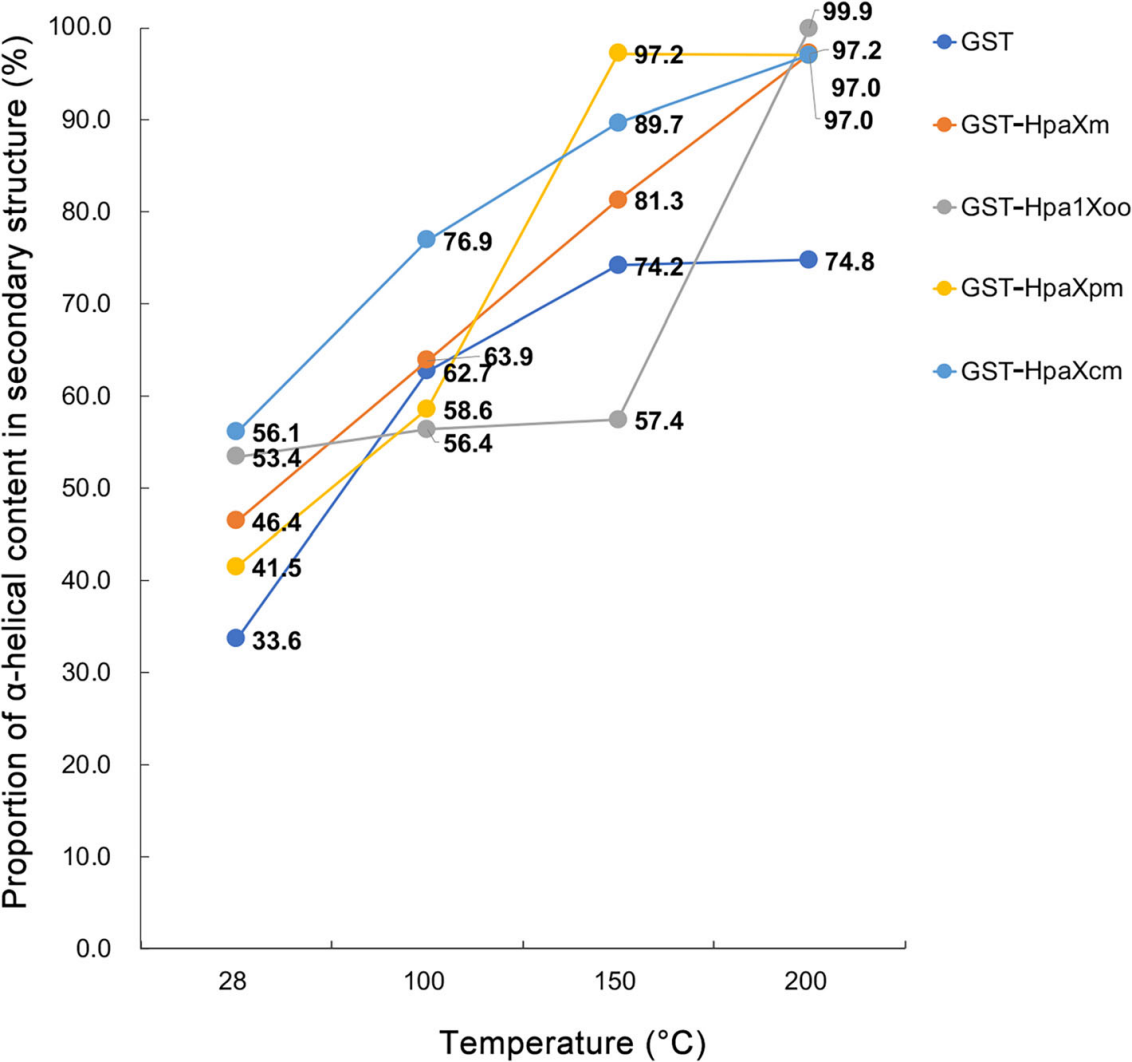
The proportion of α-helical content in secondary structure measured by CD spectra. Proportion of α-helical content in secondary structure of the four GST-Hpa1 after heat treatment at different temperaturs (28 °C, 100 °C, 150 °C, or 200 °C), with GST as a reference

Since the number of α-helices is related to the stability of the CC structure, the α-helical content in the secondary structure of the four Hpa1 and GST proteins at the four different temperatures were investigated by CD spectra (Additional file 2). The results shown that the α-helical content increased with increasing temperature up to 200 °C (Fig. 4). Specifically, at 28 °C, the secondary structure of GST-HpaXm comprised 46.4% α-helices; GST-Hpa1Xoo comprised 53.4% α-helices; GST-HpaXpm comprised 41.5% α-helices; and GST-HpaXcm comprised 56.1% α-helices; GST comprised 33.6% α-helices. The α-helical content of GST-HpaXm, GST-Hpa1Xoo, GST-HpaXpm, and GST-HpaXcm are all higher than that of GST, which is consistent with the peak results at 28 °C. At 200 °C, the α-helical content of HpaXm, Hpa1Xoo, HpaXpm, HpaXcm, and GST increased to approximately 97.2, 99.9, 97.0, 97.0, and 74.8%, respectively, compared with the results observed at 28 °C. Interestingly, at 200 °C, apart from GST, the secondary structures of the four Hpa1 were almost entirely α-helices. These discoveries indicated that the increase in temperature makes the secondary structure of the four Hpa1 tend to harbor higher numbers of α-helices.

## Discussion

Previous studies have shown that the N-terminal CC region of harpin protein is essential for eliciting HR [3, 24, 32], but the influence of CC region on the heat resistance of harpin protein is still unclear. In this study, according to the previously identified amino acid sequence of all harpin proteins [2, 4–6, 10, 12, 34–47], CC structures in harpin proteins were predicted in various plant bacterial pathogens: *Xanthomonas*, *Pseudomonas*, *Erwinia*, and *Ralstonia* in Additional file 3. Our predictions suggested that harpins from these bacteria contained at least one region that can form a CC structure. CC structures formed by harpins from bacteria belonging to the same genus were almost identical. For instance, harpins from *Erwinia*, *Ralstonia*, or *Pseudomonas* had multiple CC structure formation (CSF) regions that were evenly distributed in their protein sequences. By contrast, harpins from *Xanthomonas* had only one CSF at their N terminus, except Hpa1Xoc from *X. oryzae* pv. *oryzicola*. Although there are two predicted CSF regions in Hpa1Xoc, the main CSF is at the N-terminus. These results indicated that the four Hpa1 from *Xanthomonas*, HpaXm (*Xanthomonas citri* subsp. *malvacearum*), Hpa1Xoo (*X. oryzae* pv. *oryzae*), HpaXpm (*X. phaseoli* pv. *manihotis*), and HpaXcm (*X. citri* pv. *mangiferaeindicae*), harboring one CSF region were suitable for studying the relationship between the thermal stability of harpin and the stability of the CC structure. Therefore, in this study, we used these four Hpa1 to study the effect of the CC region near to the N-terminus of the harpin protein on their heat resistance.

To our knowledge, there has been no research prior to our study into the relationship between the stability of the CC structure and the heat resistance of the harpin protein. In this study, heat resistance assays using Hpa1 carrying aa residue mutations at the *a*, *b*, or *d* positions showed that an increase in the stability of the CC structure can lead to the higher thermal stability of Hpa1. For the first time, we proposed that the stability of CC structure at the N-terminus is positively correlated with the heat resistance of Hpa1. In general, the stability of the CC structure is determined mainly by interactions between helical chains within the same hydrophobic core [25, 29–31]. Changes to the hydrophobic interactions between aa residues at the *a*, *b*, *d*, or *g* positions can change the CC formation probability and, hence, the stability of the CC structure. For example, Ji et al. (2011) mutated the aa residue at the *a, d, e*, or *g* of hpa1Xoo from *X. oryzae* pv. *oryzae* to alter the formation of CC and found that N-terminal CC region is essential for eliciting HR, but C-terminus is not. In many studies, the ability of harpin proteins to induce HR was determined by the ratio between the size of the necrotic lesion caused by the HR and the injection site [2, 34]. Therefore, we mutated the Hpa1 aa residue at the *a*, *b*, or *d* positions to increase or decrease the PFC, and compared the HR activity of wild Hpa1 and Hpa1 mutants after different temperature treatments to determine their heat resistance. We found that the N-terminal α-helical region of all four Hpa1 was able to form CC structures, and the α-helical content increased with increasing temperature up to 200 °C, and the secondary structures of 200 °C-treated Hpa1 are all α-helices. The number of α-helices has been reported to be important for CC structural stability [29], because more helices can provide a broader hydrophobic interface which are better for shielding the core region and for polar and ionic interactions involving the residues at the b and c positions [29, 48]. Therefore, the 200 °C-treated harpin proteins were still capable of inducing HR in tobacco, probably owing to the increase in the number of α-helices, which resulted in a better shielding of the CC core.

## Conclusions

In summary, our results shown that the number of α-helices in harpin proteins and the CC formation probability are important for the heat resistance of these proteins. And the number of α-helices in harpin proteins and the CC formation probability are two major determinants for the stability of the CC structure. Therefore, we concluded that the stability of the CC structure is a key factor controlling the heat resistance of harpin proteins.

## Supplementary Information

**Additional file 1.** Predictions of the probability of forming a Coiled-coil structure of harpin proteins.

**Additional file 2.** Western blot analysis of efficient expression of Hpa1, and their mutants. Wildtype Hpa1, their mutants and GST (negative control) were probed with a polyclonal antibody specific for GST and then a goat anti-rabbit lgG-HRP antibody. The induced bacterial cultures were ultra-sonicated followed by centrifugation. The resulting supernatants were used as soluble protein samples. M, molecular markers. Molecular mass marker in size (kDa) is indicated on the left-hand side.

**Additional file 3.** Secondary structural changes of four Hpa1 proteins under four different temperatures. (A) CD spectra of four Hpa1 proteins measured after 10 min incubation at 28 °C. (B). Secondary structural contents (%) of four Hpa1 proteins after temperature treatment at 28 °C, 100 °C, 150 °C, or 200 °C. Secondary structural contents were calculated using Jasco’s Spectra. Manager TM software. Detailed results are presented in the table on the right-hand side. The α-helical content of the four Hpa1 and GST proteins after treatment at 200 °C treatment is shown in red.

## Data Availability

All data generated or analyzed during this study are included in this published article.

## Abbreviations

HR: Hypersensitive response
CC: Coiled-coil
3-D: Three dimensional
PFC: The probability of forming a CC
CSF: The coiled-coil structure formation
FDM: Fast Site-Directed Mutagenesis
CD: Circular dichroism
