# Supplementary material for: The stability of the coiled-coil structure near to N-terminus influence the heat resistance of harpin proteins from Xanthomonas
Source: BMC Microbiol. 2020 Nov 12;20:344. doi: 10.1186/s12866-020-02029-6 (PMC7663895; doi:10.1186/s12866-020-02029-6)
Supplement: Supplementary file 1 — Additional file 1. Predictions of the probability of forming a Coiled-coil structure of harpin proteins. [file 12866_2020_2029_MOESM1_ESM.pdf]

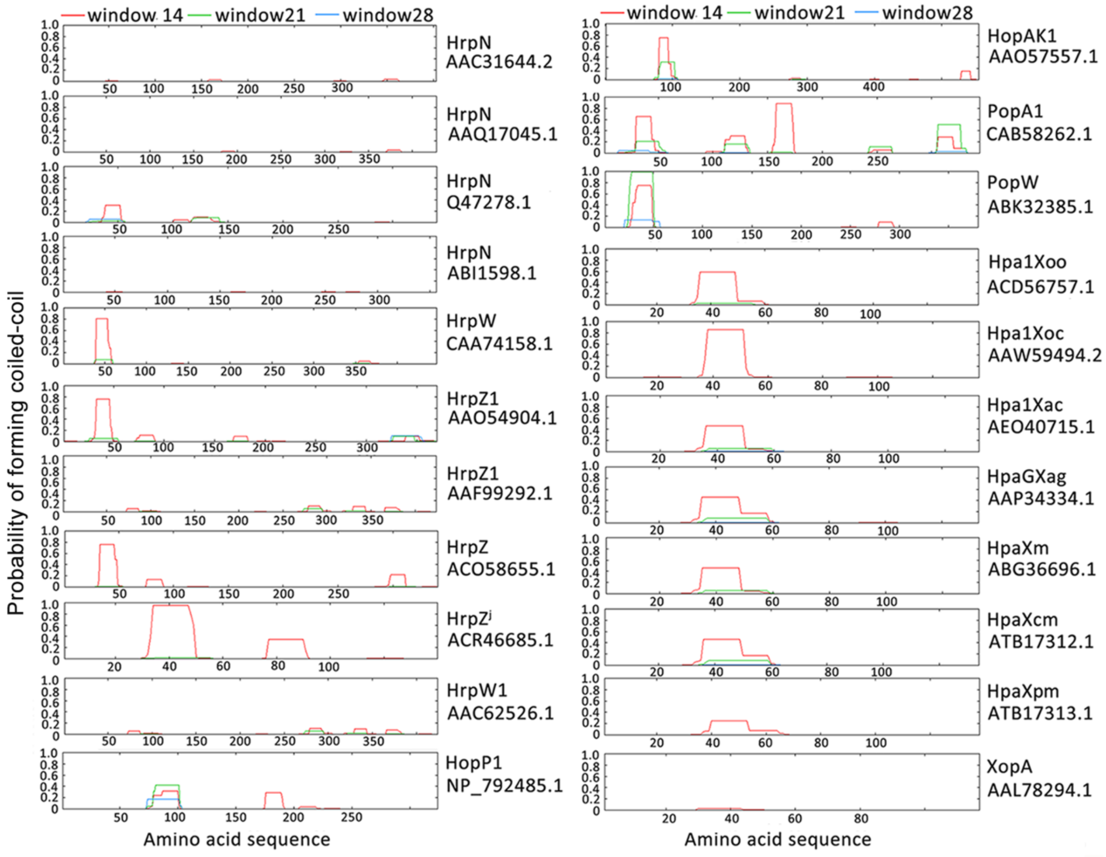

Additional file 1. Predictions of the probability of forming a Coiled-coil structure of harpin proteins.
