# Supplementary material for: The stability of the coiled-coil structure near to N-terminus influence the heat resistance of harpin proteins from Xanthomonas
Source: BMC Microbiol. 2020 Nov 12;20:344. doi: 10.1186/s12866-020-02029-6 (PMC7663895; doi:10.1186/s12866-020-02029-6)
Supplement: Supplementary file 2 — Additional file 2. Western blot analysis of efficient expression of Hpa1, and their mutants. Wildtype Hpa1, their mutants and GST (negative control) were probed with a polyclonal antibody specific for GST and then a goat anti-rabbit lgG-HRP antibody. The induced bacterial cultures were ultra-sonicated followed by centrifugation. The resulting supernatants were used as soluble protein samples. M, molecular markers. Molecular mass marker in size (kDa) is indicated on the left-hand side. [file 12866_2020_2029_MOESM2_ESM.pdf]

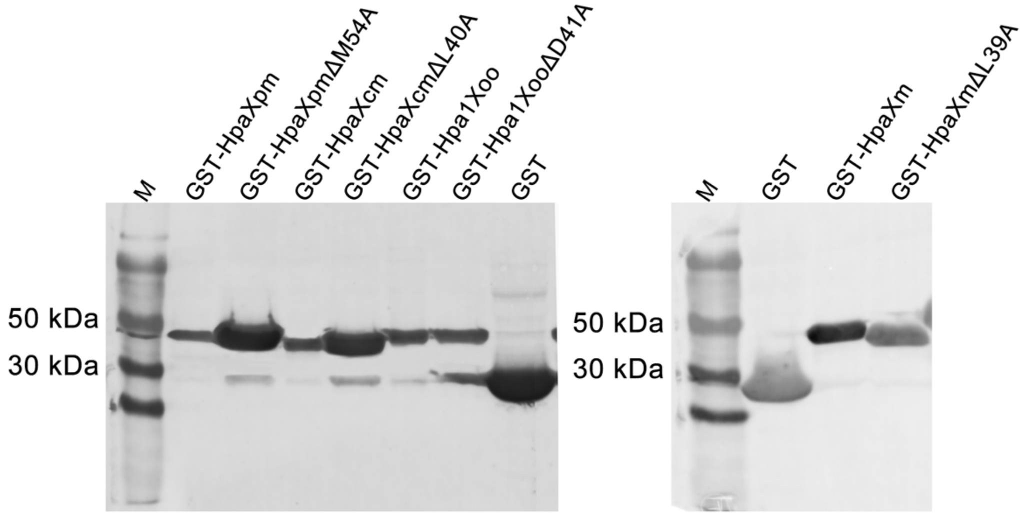

Addtional file 2. Western blot analysis of efficient expression of Hpa1, and their mutants. Wild-type Hpa1, their mutants and GST (negative control) were probed with a polyclonal antibody specific for GST and then a goat anti-rabbit lgG-HRP antibody. The induced bacterial cultures were ultra-sonicated followed by centrifugation. The resulting supernatants were used as soluble protein samples. M, molecular markers. Molecular mass marker in size (kDa) is indicated on the left-hand side.
