# Supplementary material for: The stability of the coiled-coil structure near to N-terminus influence the heat resistance of harpin proteins from Xanthomonas
Source: BMC Microbiol. 2020 Nov 12;20:344. doi: 10.1186/s12866-020-02029-6 (PMC7663895; doi:10.1186/s12866-020-02029-6)
Supplement: Supplementary file 3 — Additional file 3. Secondary structural changes of four Hpa1 proteins under four different temperatures. (A) CD spectra of four Hpa1 proteins measured after 10 min incubation at 28 °C. (B). Secondary structural contents (%) of four Hpa1 proteins after temperature treatment at 28 °C, 100 °C, 150 °C, or 200 °C. Secondary structural contents were calculated using Jasco’s Spectra. Manager TM software. Detailed results are presented in the table on the right-hand side. The α-helical content of the four Hpa1 and GST proteins after treatment at 200 °C treatment is shown in red. [file 12866_2020_2029_MOESM3_ESM.pdf]

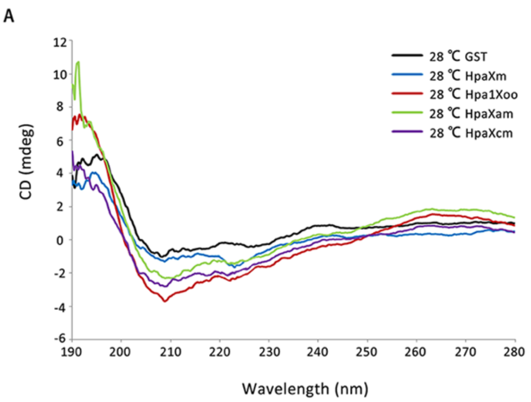

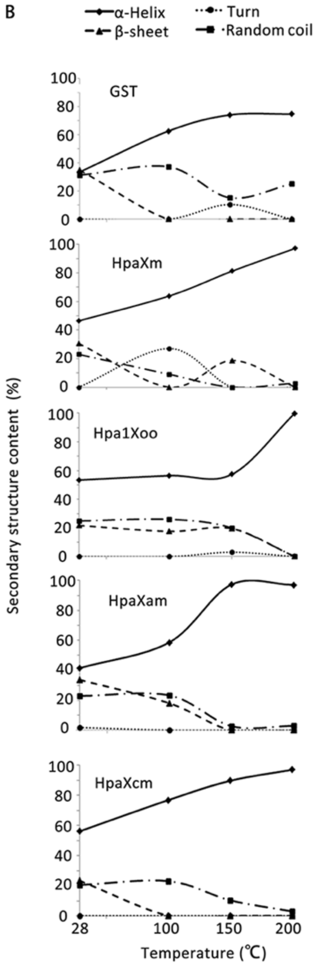

Protein	Temperature (℃)	Secondary structure content (%)			
		α-helix	β-sheet	Turn	Random coil
GST	28	33.6	35.0	0	31.4
	100	62.7	0	0	37.3
	150	74.2	0	10.5	15.3
	200	74.8	0	0	25.2
HpaXm	28	46.4	30.6	0	23.0
	100	63.9	0	27.1	9.0
	150	81.3	18.7	0	0
	200	97.2	0	0	2.8
Hpa1Xoo	28	53.4	21.7	0	24.9
	100	56.4	17.6	0	26.0
	150	57.4	19.5	3.1	20.0
	200	99.9	0	0	0.1
HpaXam	28	41.5	33.7	1.9	22.9
	100	58.6	18.1	0	23.3
	150	97.2	0	0	2.8
	200	97.0	0	0	3.0
HpaXcm	28	56.1	23.4	0	20.5
	100	76.9	0	0	23.1
	150	89.7	0	0	10.3
	200	97.0	0	0	3.0

Additional file 3. Secondary structural changes of four Hpa1 proteins under four different temperatures. (A) CD spectra of four Hpa1 proteins measured after 10 min incubation at 28°C. (B) Secondary structural contents (%) of four Hpa1 proteins after temperature treatment at 28°C, 100°C, 150°C, or 200°C. Secondary structural contents were calculated using Jasco's Spectra Manager ™ software. Detailed results are presented in the table on the right-hand side. The α-helical content of the four Hpa1 and GST proteins after treatment at 200°C treatment is shown in red.
